# Electrocardiography Assessment of Sympatico–Vagal Balance during Resting and Pain Using the Texas Instruments ADS1299

**DOI:** 10.3390/bioengineering10020205

**Published:** 2023-02-03

**Authors:** Donghua Liao, Rasmus B. Nedergaard, Misbah Unnisa, Soumya J. Mahapatra, Mahya Faghih, Anna E. Phillips, Dhiraj Yadav, Vikesh K. Singh, Søren S. Olesen, Rupjyoti Talukdar, Pramod K. Garg, Imran K. Niazi, Christina Brock, Asbjørn M. Drewes

**Affiliations:** 1 Mech-Sense, Department of Gastroenterology and Hepatology, Aalborg University Hospital, 9000 Aalborg, Denmark; 2 Department of Medical Gastroenterology, Asian Institute of Gastroenterology, Hyderabad 500082, India; 3 Department of Gastroenterology, All India Institute of Medical Sciences, New Delhi 110016, India; 4 Division of Gastroenterology, Department of Medicine, Johns Hopkins Medical Institutions, Baltimore, MD 21287, USA; 5 Pancreatitis Center, Department of Medicine, Johns Hopkins Medical Institutions, Baltimore, MD 21287, USA; 6 Division of Gastroenterology, Hepatology, and Nutrition, Department of Medicine, School of Medicine, University of Pittsburgh, Pittsburgh, PA 15213, USA; 7 Department of Clinical Medicine, Aalborg University, 9220 Aalborg, Denmark; 8 Centre for Chiropractic Research, New Zealand College of Chiropractic, Auckland 1060, New Zealand

**Keywords:** sympatico–vagal balance, electrocardiography (ECG), biomarkers, cold pressor test

## Abstract

Sympatico–vagal balance is essential for regulating cardiac electrophysiology and plays an important role in arrhythmogenic conditions. Various noninvasive methods, including electrocardiography (ECG), have been used for clinical assessment of the sympatico–vagal balance. This study aimed to use a custom-designed wearable device to record ECG and ECG-based cardiac function biomarkers to assess sympatico–vagal balance during tonic pain in healthy controls. Nineteen healthy volunteers were included for the ECG measurements using the custom-designed amplifier based on the Texas Instruments ADS1299. The ECG-based biomarkers of the sympatico–vagal balance, (including heart rate variability, deceleration capacity of the heart rate, and periodic repolarization dynamic), were calculated and compared between resting and pain conditions (tonic pain). The custom-designed device provided technically satisfactory ECG recordings. During exposure to tonic pain, the periodic repolarization dynamics increased significantly (*p* = 0.02), indicating enhancement of sympathetic nervous activity. This study showed that custom-designed wearable devices can potentially be useful in healthcare as a new telemetry technology. The ECG-based novel biomarkers, including periodic repolarization dynamic and deceleration capacity of heart rate, can be used to identify the cold pressor-induced activation of sympathetic and parasympathetic systems, making it useful for future studies on pain-evoked biomarkers.

## 1. Introduction

The cardiac autonomic nervous system consists of two branches, the sympathetic and parasympathetic systems, that work in a delicately tuned, yet opposing fashion in several organs including the heart [1]. Assessing the sympatico–vagal balance in routine clinical or experimental conditions has always been a primary goal for cardiac risk stratification methods. Various parameters, including heart rate variability (HRV) [2,3], baroreflex sensitivity [4,5,6], deceleration capacity (DC) of heart rate [7], and periodic repolarization dynamic (PRD) [3,8,9,10], have been employed to determine the individual level of sympatico–vagal balance. Compared to the HRV and baroreflex, the PRD provides a measurement of the sympathetic effect on cardiac repolarization to reflect influences at the level of the left ventricular myocardium [8]. On the other hand, the DC is based on the advanced phase-rectified signal averaging method, allowing for an isolated analysis of oscillations associated with a deceleration of heart rate, and thus mainly reflects the vagal branch activity of the cardiac autonomic nervous system [3,7,11,12].

The cold pressor test is a method for tonic pain stimulation where the subjects immerse their hand in ice water. The pain stimulation activates the autonomic nervous system and influences the sympatico–vagal balance. It has been suggested that the co-activation between the sympathetic and vagal activity results in inconsistent HRV indices in several cold pressor test studies [13,14,15,16]. In contrast, others reported that tonic cold pain results in sympathetic activation assessed as altered cardiac function in terms of changes in HRV and increased blood pressure [5,6,14]. The variable changes in the HRV parameters could be attributed to the opposite changes in cardiac autonomic regulation between the sympathetic and parasympathetic systems [15]. Hence, novel parameters independent of the HRV indices are needed to evaluate the individual cold pressor-induced sympatico-vagal balance.

Recently, Rizas et al. have proposed PRD as an electrocardiogram (ECG)-based risk predictor. The PRD has been suggested to reflect sympathetic-associated effects on ventricular myocardium by measuring low-frequency (below 0.1 Hz) oscillations of cardiac repolarization instability [8,9,17]. The PRD occurs independently of respiratory activity in humans when comparing respiratory rates of 10 and 20/min with constant minute ventilation [18]. Moreover, the PRD has been confirmed not to be an epiphenomenon of HRV parameters [4,18]. However, the effect of the cold pressor condition on the PRD change is yet unknown.

The present study aimed to (1) investigate the feasibility and signal quality of the ECG recordings on a new custom-designed wearable ECG and electroencephalographic (EEG) combined device and (2) use HRV indices in time and frequency domain, DC of heart rate, and PRD to assess the effect of a short-term cold pressor test on cardiac electrical function and the sympatico–vagal balance in healthy volunteers. We hypothesized that the cold pressor test would increase sympathetic drive with increased PRD compared to a resting condition.

## 2. Materials and Methods

### 2.1. Data Recording System

**Figure 1:**
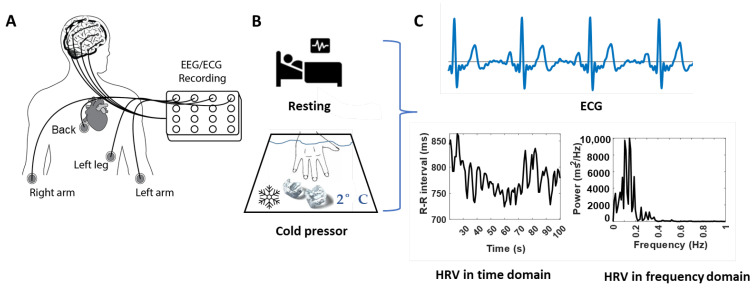
Demonstration of the experimental protocol and representative HRV characteristics from ECG signals. (**A**): Schematic drawing of the combined two 8-channel ADS1299 systems and the ECG electrodes positions. (**B**): Schematic drawing of the tests at the baseline/resting (top panel) and the tonic pain/cold pressor (bottom panel) conditions. (**C**): Standard ECG signals (top panel) and the calculated HRV behavior (bottom panels) in time (left panel) and frequency (right panel) domains.

This study used a custom-built wearable system including two 8-channel Texas Instruments ADS1299 amplifiers combined to create a 16-channel recording platform. The system was thoroughly evaluated on EEG recordings by comparing it to a high quality laboratory-based system (NuAmps, Compumedics Neuroscan, Dresden, Germany) [19]. Compared to the laboratory-based system, the custom-built system showed no significant differences in both EEG-specific measures (such as power across bands, power ratio across bands, and pre-movement noise), and movement-related cortical potential specific measures (such as signal-to-noise ratio as well as time and amplitude of the negative peak) [19]. In this study, we developed the system to simultaneously record 9 channels of EEG and 4 channels of ECG (Figure 1A).

### 2.2. Participants

The study was carried out at the Department of Medical Gastroenterology, Asian Institute of Gastroenterology, Hyderabad; and the Department of Gastroenterology, All India Institute of Medical Sciences, New Delhi, India. The study was approved by the Institutional Review Board of All India Institute of Medical Sciences, New Delhi, India (ref. no. IECPG-670/25.11.2020). Nineteen healthy participants (10 participants from the Asian Institute of Gastroenterology, Hyderabad; and 9 from All India Institute of Medical Sciences, New Delhi, (average age: 28.0 ± 0.9 years, 36% female) were recruited through professional networks. Participants were excluded if they had a history of any neurological disorders, cardiac disorders, hypertension, medications, or epilepsy. All participants gave written informed consent before enrollment.

### 2.3. Experiment Procedures

Experimental procedures were identical for all participants, seated comfortably, and instructed to focus on a fixed point at the wall and minimize body movements during ECG and EEG recordings. Each subject underwent two separate ECG and EEG recordings: 2 min of ECG and EEG recorded at rest and during the cold pressor test. During this test, they were instructed to position their nondominant hand in a cold-water bath of 2 °C for two minutes or as long as they could tolerate it (Figure 1B). ECG and EEG data were collected, and we used only ECG data for the following data analyses in this study.

The four ECG channels were connected via the electrodes placed on the left arm, right arm, left leg, and back to configure the Wilson Tetrahedron 4-leads ECG system [20,21] (Figure 1A). During the recordings, the reference and ground channels used for EEG and ECG recordings were located at the ref and gd locations for the OpenBCI EEG Electrode Cap Kit.

### 2.4. Data Processing

ECG data in csv format were preprocessed offline on MATLAB using a combination of custom subroutines (R2022a, MathWorks, Inc., Natick, MA, USA) and EEGLAB (SCCN, Institute for Neural Computation, University of California San Diego, San Diego, CA, USA) as follows:

1. Building zero-phase shift bandpass filter (2–26 Hz);
2. Zero-phase filtering using the built band-pass filter in 1) and the scale values = 1;
3. Visual inspection of data quality, the offset of the isoline of the ECG data was estimated and removed;
4. Rereferencing to the common average reference.

The filtered ECG data were then used for the following calculations on vectorcardiography vectors and ECG-based biomarkers analysis.

#### 2.4.1. Vectorcardiography Vectors

The exported surface ECG signals in the Wilson system were transformed into a spatial vectorcardiography system [22] using the customized MATLAB subroutines. The vectorcardiography system is an anatomical plane-based three-dimensional orthogonal system with three components: *X*-axis (right-to-left) component *V_x_*, *Y*-axis (head-to-foot) component *V_y_*, and *Z*-axis (front-to-back) component *V_z_*. The *V_x_*, *V_y_*, and *V_z_* can be converted from the recorded surface electrode signals in Wilson Tetrahedron ECG system as [20,21],

(1) $$\left\{\begin{array}{c} V_{x}\\ V_{y}\\ V_{z} \end{array}\right\}=3\times K\times \left\{\begin{array}{c} p_{x}\\ p_{y}\\ p_{z} \end{array}\right\}$$

where *V_x_, V_y_,* and *V_z_* are voltage components in *X*-, *Y*-, and *Z*- axis in the vectorcardiography system, $p_{x},p_{y},\;and\;p_{z}$ are the direction cosines of the dipole vector, *K* $=1/3\left(\sqrt{\frac{3}{8}\times E^{2}+V_{B}^{2}}\right)$, $E^{2}={\left(V_{L}-V_{R}\right)}^{2}+1/3{\left[\left(V_{F}-V_{R}\right)+\left(V_{F}-V_{L}\right)\right]}^{2}$ is a constant of proportionality. *V_R_, V_L_, V_F_,* and *V_B_* are the potentials of the electrodes recorded in the Wilson system at the right arm, left arm, left leg, and back. $p_{x},p_{y},\;and\;p_{z}$ in Equation 2 can be calculated from the recorded potentials of the electrodes as [21]:

(2) $$\left\{\begin{array}{c} vp_{x}\\ vp_{y}\\ vp_{z} \end{array}\right\}=\left[\begin{array}{ccc} 122 & 27 & -20\\ -26 & 55 & 15\\ -30 & -52 & 139 \end{array}\right]\left\{\begin{array}{c} V_{L}-V_{R}\\ V_{F}-V_{R}\\ V_{B}-V_{R} \end{array}\right\},\;and\;\left\{\begin{array}{c} p_{x}\\ p_{y}\\ p_{z} \end{array}\right\}=\left\{\begin{array}{c} vp_{x}/\sqrt{vp_{x}^{2}+vp_{y}^{2}+vp_{z}^{2}}\\ vp_{y}/\sqrt{vp_{x}^{2}+vp_{y}^{2}+vp_{z}^{2}}\\ vp_{z}/\sqrt{vp_{x}^{2}+vp_{y}^{2}+vp_{z}^{2}} \end{array}\right\}\;\;\;$$

#### 2.4.2. ECG-Based Biomarkers

With the obtained vectors *V_x_*, *V_y_*, and *V_z_*, the QRS boundaries, T-wave peak, and T-wave offset were detected and synchronized in a beat-to-beat manner using the modified open-source software ECGdeli [23]. With the detected QRS complex and T-wave boundaries, the parameters, including HRV, DC of the heart rate, and PRD, can be calculated.

- *HRV parameters*

The HRV parameters, including RR intervals, heartbeat rate (HR), low-frequency power (LF), high-frequency power (HF), and LF/HF ratio, were performed in the time or frequency domains as described in Malik et al. [2] (Figure 1C). The time-domain variables include the RR intervals and HR, where the RR intervals are the mean intervals between adjacent QRS complexes resulting from sinus node depolarizations. The RR intervals and HR were calculated based on the detected R-peaks over time, where the artifacts and ectopic beats were removed. The beats were considered ectopic if the preceding RR interval differed by more than 20% from the last valid RR interval. The frequency-domain variables, including LF (0.04–0.15 Hz), HF (0.15–0.4 Hz), and LF/HF ratio, were calculated by integrating the power spectral density curves. The power spectral density curves were computed using the fast Fourier transform algorithm from the RR intervals time sequences.

- *Deceleration capacity*

The DC of the heart rate was measured from beat-to-beat RR interval time series using the phase-rectified signal averaging method described in Bauer et al. [7,11] as:

1. An anchor point, defined as the heartbeat intervals longer than the preceding interval.
2. Windows of 2 L values, defined around each anchor point, where L is the point number previous and posterior to the anchor point in the RR interval curve. Anchor points in the last L samples of the RR intervals were discarded, as windows of length 2 L could not be defined around them. In this study, L = 12 was chosen because it was the minimum value to detect the low frequencies of the RR intervals series in the range of interest (0.04–0.15) Hz.
3. The phase-rectified signal averaging series was obtained by averaging the RR values over all 2 L-sample windows contained in recordings during resting and cold pressor.
4. A DC value was calculated from the phase-rectified signal averaging series at the anchors $X\left(0\right)$, the point immediately following the anchors $X\left(1\right)$, and the two points preceding the anchors $X\left(-1\right)$ and $X\left(-2\right)$ as:
  (3) $$\;DC=\left[X\left(0\right)+X\left(1\right)-X\left(-1\right)-X\left(-2\right)\right]/4$$

- *Periodic repolarization dynamics*

The assessment of PRD was based on the dynamic change of the angles between two consecutive T-waves of the obtained vectorcardiography vectors. The T-wave angle was used as an estimate of the instantaneous repolarization instability [3,9,10,17].

In this study, the T-waves were first detected automatically using ECGdeli program. However, in some cases, the detected T-waves were inaccurate as T-waves, and P-wave amplitudes of *V_y_* were too low to be distinguished from each other, or the T-wave inversion in *V_z_* was misidentified. Then the T-waves were defined from the detected QRS complex as described in [24]:

For each beat *i*, *RRi* is the RR interval of the beat, and the T-wave window onset, denoted by *Toni*, was set at 90 ms after the QRSi mark: *Toni = QRSi + 90 ms*. The T-wave window end, denoted by *Tendi*, was defined as *Tendi = QRSi + min(360 ms, 2/3RRi)* for *RRi* below 720 ms. For *RRi* equal to or higher than 720 ms, *Tendi = QRSi + 360 ms.*

With the detected T-waves in the vectorcardiography system, the angle dT° between two consecutive T-waves was calculated by using the dot product of each pair of consecutive average T-wave vectors [3,9,10,17]. The PRD calculation was conducted after filtering the dT° time series using a 10th-order median filter.

In this study, the phase-rectified signal averaging based PRD calculation was used for the assessment of the PRD in resting and cold pressor conditions as [10,17,24]:

1. The phase-rectified signal averaging of the dT° time series was obtained using a similar procedure to the above phase-rectified signal averaging estimations in deceleration capacity calculation. The anchor points were defined by comparing averages of *M = 9* values of the dT° series previous and posterior to the anchor point candidate (*x_i_*). A beat *i* is considered an anchor point if:

(4) $$\frac{1}{M}\overset{M-1}{\underset{j=0}{\sum }}x_{i+j}>\frac{1}{M}\overset{M}{\underset{j=1}{\sum }}x_{i-j}$$

2. Windows of 2 L values were defined around each anchor point. In this study, L = 20 was chosen because it was the minimum value to detect frequencies in the range of interest (0.025–0.1) Hz, as described in Palacios [10].
3. Phase-rectified signal averaging series were obtained by averaging the dT° series overall defined windows.
4. PRD was defined as the difference between the maximum and minimum values of the phase-rectified signal averaging series.

### 2.5. Statistical Analysis

Data are presented as mean ± SE. The Shapiro–Wilk normality test was first employed to test the normal distribution of the data. The Student *t*-test or Mann–Whitney U test were used to compare data between two institutions for normally or nonnormally distributed parameters. Paired *t*-tests were used for comparing ECG-based biomarkers between resting and cold pressor conditions. *p*-values < 0.05 were considered statistically significant. All statistical analyses were carried out using SPSS (version 27.0).

## 3. Results

All participants fully complied with the study protocol. Datasets from two participants were excluded due to the invalid datasets induced by the loose connections of the ECG electrode on the right arm for one participant and the poor signal-to-noise ratio for another participant. Only the ECG recordings at the resting state were used for one participant due to the muscle tremor artifacts induced by the cold pressor test. Data from each participant are shown in Table S1 in the supplementary material. Comparing the data from two different institutions, the DC during the cold pressor test from All India Institute of Medical Sciences, New Delhi, was higher than that from the Asian Institute of Gastroenterology, Hyderabad (*p* = 0.04). There were no differences between the two institutions for HRV parameters and PRD in resting and cold pressor conditions and the endurance time in cold water (*p* > 0.13).

### 3.1. ECG Signals

**Figure 2:**
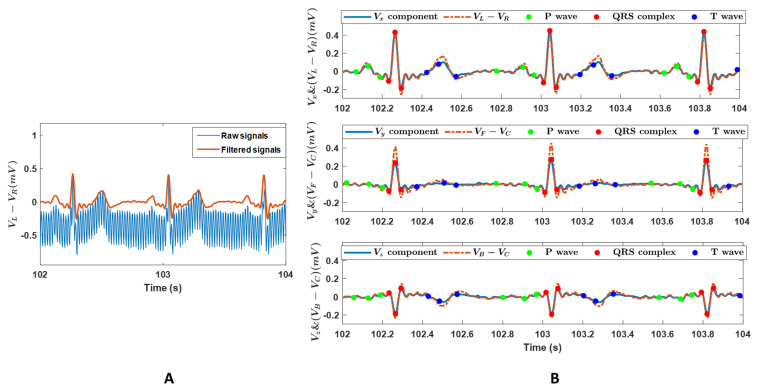
(**A**): A representative example of the raw and the filtered ECG signals. Data are voltage differences between the left and right arm (*V*_L_ − *V*_R_). (**B**): The voltage components in the *X*-, *Y*-, and *Z*- axis in the vectorcardiography system, *V_x_*, *V_y_*, and *V_z_*, and the voltage differences of (*V*_L_ − *V*_R_), (*V*_F_ − *V*_C_), and (*V*_B_ − *V*_C_). *V*_R_, *V*_L_, *V*_F_, and *V*_B_ are recordings on the right arm, left arm, left leg, and back in the Wilson system, and *V*_C_ = (*V*_R_ + *V*_L_ + *V*_F_)/3. The components *V_x_*, *V_y_*, and *V_z_*, were converted from the datasets in panel A. Scatters are the detected *p*-waves, QRS complexes, and T-waves.

Figure 2A shows a representative example of the recorded raw and filtered ECG signals. It showed the oscillations and offsets of the signal were successfully removed after the filtering. Figure 2B shows the *V_x_, V_y_,* and *V_z_* components converted from the dataset presented in Figure 2A, the detected *p*-waves, QRS complexes, T-waves, and the corresponding voltage differences calculated from the surface ECG signals. The ECG signals in Figure 2A,B showed a typical pattern of an electrogram of the heart, where the three main components of an ECG, including *p*-wave, QRS complex, and T-wave, can be identified, confirming the feasibility and signal quality of the recordings. The identified waveforms in Figure 2B approved the detection accuracy of the selected open-source software ECGdeli.

### 3.2. ECG-Based Cardiac Function Biomarkers

**Figure 3:**
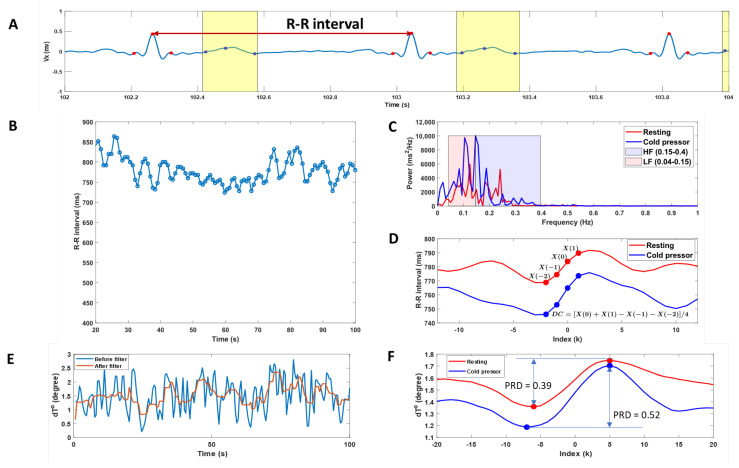
A single representative example of the calculated heart rate variability (HRV), deceleration capacity of heart rate (DC), and periodic repolarization dynamics (PRD). (**A**): Vx component of the ECG signals with detected QRS complexes (red scatters) and T-waves (blue scatters and the yellow box). (**B**): Heartbeat (RR) intervals change during the experimental period. (**C**): Spectral analysis in the frequency domain of the RR interval data during resting and cold pressor test. The low-frequency power (0.04–0.15 Hz) and high-frequency power (0.15–0.40 Hz) were assessed by integrating the power spectral density curves. (**D**): Deceleration-related phase-rectified signal averaging curves of the heartbeat (RR) intervals during resting and cold pressor tests. The DC of heart rate was calculated from phase-rectified signal averaging curves of the RR intervals at index points of 0, 1, −1, and −2. (**E**): dT° angle change over time before and after low-pass filtering. (**F**): Phase-rectified signal averaging curves of the dT° angle during resting and cold pressor tests. The magnitude of the oscillations is quantified using PRD, which is a measure of the amplitude of the central part of the phase-rectified signal averaging curves.

Figure 3 shows a single representative example for the calculated HRV parameters in time and frequency domains, DC of heart rate, and PRDs in resting and cold pressor conditions.

The averaged ECG-based biomarkers, including HRV parameters, DC of heart rate, and PRD, during resting and cold pressor conditions, are shown in Table 1, where the results from the individual participant are shown in Table S1 in the supplementary material.

Neither the HRV parameters nor DC showed a difference between the resting and cold pressor conditions. The cold pressor test significantly increased PRD (*p* = 0.02), indicating increased sympathetic responses (Figure 3F).

## 4. Discussion

The present study showed that the custom-designed wearable ECG and EEG combined system can provide technically satisfactory ECG recordings. To the best of our knowledge, the present study is the first to assess the effect of a short-term cold pressor test on sympatico–vagal balance using two novel ECG-based biomarkers: the DC of heart rate and PRD. We observed that exposure to tonic cold pain significantly increased PRD, reflecting an enhancement of sympathetic activity.

The PRD increase induced by the cold pressor is in line with previous studies during cold exposure, where the T-wave characteristics, including T-peak to T-end interval and T-wave amplitude (directly associated with the PRD calculations), were increased [14]. However, compared to previous time interval-based T-wave characteristics, the PRD is superior by integrating all the spatiotemporal information of the T-wave into T-wave vectors, allowing a more robust characterization of beat-to-beat repolarization variations to anticipate, e.g., ventricular repolarization heterogeneity in cardiac arrhythmias [17].

Noninvasive evaluation of the autonomic control of heart rate in real-life conditions is possible through HRV analysis using different HRV indices to measure various aspects of HRV [25]. However, previous studies during whole-body cold water immersion [26], short-term cold exposure [14,16], or facial cooling [15] evidenced the co-activation of sympathetic and parasympathetic nervous systems during cold stimulations. The co-activation caused inconsistent HRV analyses where both the heartbeat rate and the changes in HRV indices appeared highly variable on an individual subject basis [13,14,15,16]. Similar findings were observed in the current study where, compared to the rest state, seven of seventeen (41%) subjects had about 5% longer RR intervals during the cold pressor test, indicating parasympathetic drive (Table S1). The co-activation of the sympathetic and parasympathetic nervous systems during the cold stimulations in these seven subjects might explain why there were no differences in any HRV parameters between the cold pressor test and the resting conditions in this study. In addition to the longer RR intervals for these seven subjects in the cold pressor test, another interesting finding worth noting was that the DC of heart rate for these seven subjects was also higher during the cold pressor condition (Table S1). Since DC of heart rate was believed to mainly reflect the vagal branch of the cardiac autonomic nervous system [3,7,12], the finding may imply the enhanced activation of the parasympathetic nervous system during cold stimulations in these subjects. However, those findings were based on the limited number of participants in this study. A full-scale investigation is needed to confirm the associations between the prolonged RR intervals and the DC of heart rate change during the cold pressor condition. Sympathovagal co-activation of the autonomic nervous system is a regulatory process that may serve as a protective cardiovascular effect but could change heartbeat rate dynamics from more fractal to random heartbeat rate organization [15] and predispose to arrhythmias [26]. Hence, the analysis with DC of heart rate and PRD combination could shed some light on quantitative identification of the co-activated sympathetic and efferent parasympathetic nervous systems during challenge tests such as the cold pressor condition.

This study used the developed custom-designed ADS1299 system for ECG signals [19]. Compared to the previous version of the system, the updated version combined the EEG and ECG recording in the design, allowing the assessment of cardiac and neural activity simultaneously [27]. This study is the first to use this custom-designed system to simultaneously record ECG and EEG signals. As the EEG recordings from the system were previously validated [19], only the ECG data were investigated in this study to demonstrate the signal quality of these recordings. With the confirmed signal quality of the ECG recordings, the simultaneously recorded ECG and EEG signals can be further used to assess the brain and autonomic nervous system responses to pain stimulation as an extension to quantitative sensory testing [28]. The custom-designed EEG and ECG combined amplify system is limited to allow a maximum of four channels that can be used for the ECG recordings. However, the PRD calculation must be done in a spatial vectorcardiography ECG system, which is why Wilson’s 4-leads ECG system rather than Frank’s 7-leads ECG system, the one that was previously used for PRD analysis [8,9,17], was applied in this study. Although the Wilson system is more accurate than two other 4-leads ECG systems (Duchosal-Sulzer System and Grishman-Schcrlis System) [29], previous torso tests demonstrated that the Wilson system provided inferior results in comparison to Frank’s 7-leads ECG system [30]. However, compared to the original Wilson system [20], we used the update coefficients matrix [21] to transform the Wilson ECG system into the spatial vectorcardiography system, thus improving the accuracy of the transformation. The more accurate 4-leads ECG system, such as EASI system (using Frank’s E, A, and I electrode locations, and the fourth electrode, S, lies at the upper end of the sternum) needs to be validated and considered in future investigations [31,32].

This study used the automated synchronized multichannel method for QRS and T wave peak detection [23]. The advantage of the process is that no prior knowledge about the quality of individual channels is required. However, for vectorcardiography system components *V_y_* and *V_z_* used to identify T-waves were in some cases challenging using the automatic analysis due to the low amplitudes of *p*-waves and T-waves in *V_y_* and T-waves inversion in *V_z_*. Therefore, the obtained R-peaks were used to detect the T-waves for those datasets in this study. However, these limitations could be overcome by improving the current detection program or employing a wavelet-based clustering algorithm for ECG data analysis [33]. This study used traditional HRV analysis in time and frequency domains to estimate HRV characteristics and DC of the heart rate. However, as the mechanisms involved in cardiovascular regulation probably interact with each other in a non-linear manner, conventional HRV parameters (including RR interval, HR, LF, and HF), might not be sufficient to characterize the complex dynamics of the heartbeat generation [34,35]. Thus, in future studies, nonlinear HRV indices, such as fractal-scaling exponent (predicting fatal cardiovascular events in several populations) [34,36] and approximate entropy (describing the complexity of RR interval behavior and providing information on the vulnerability to atrial fibrillation) [35] should also be considered.

## 5. Conclusions

The present study showed that the ECG and EEG combined ADS1299 system can provide technically satisfactory ECG recordings, allowing cardiac and neural activity assessment simultaneously. The system can potentially be further developed into a big ECG data-based cyber–physical system [37,38,39] in healthcare as a new telemetry technology to assess disease development and prognosis. The ECG-based novel biomarkers, including PRD and DC of heart rate, can be used to identify the cold pressor-induced activation of sympathetic and parasympathetic systems, making it useful for future studies of pain-evoked biomarkers.

## Figures and Tables

**Table 1:** The ECG-based cardiac function biomarkers in resting and cold pressor conditions.

ECG-Based Biomarkers		Resting	Cold Pressor	*p*-Values
HRV	RR intervals (ms)	800 ± 13	799 ± 14	0.35
	Heartbeat rate (bpm)	76 ± 1.2	76 ± 1.4	0.23
	LF (ms^2^)	725 ± 128	816 ± 185	0.81
	HF (ms^2^)	712 ± 116	893 ± 185	0.37
	LF/HF	1.43 ± 0.3	1.04 ± 0.3	0.36
DC (ms)		14.3 ± 1.3	16.1 ± 1.6	0.25
PRD (degree)		0.38 ± 0.01	0.42 ± 0.02	0.02 *

Notes: data are mean ± SE. *, *p* < 0.05 for comparisons between resting and cold pressor conditions. LF: low-frequency power (0.04–0.15 Hz); HF: high-frequency power (0.15–0.4 Hz); DC: deceleration capacity of heart rate; PRD: periodic repolarization dynamics.

## Data Availability

The datasets generated during and/or analyzed during the current study are available from the corresponding author upon reasonable request.

## Supplementary Materials

The following supporting information can be downloaded at: https://www.mdpi.com/article/10.3390/bioengineering10020205/s1, Table S1: The ECG-based individual cardiac function biomarkers in resting and cold pressor conditions.
