# Supplementary material for: Electrocardiography Assessment of Sympatico–Vagal Balance during Resting and Pain Using the Texas Instruments ADS1299
Source: Bioengineering (Basel). 2023 Feb 3;10(2):205. doi: 10.3390/bioengineering10020205 (PMC9952434; doi:10.3390/bioengineering10020205)
Supplement: Supplementary file 1 [file bioengineering-10-00205-s001.zip › bioengineering-2082166-supplementary.pdf]

Supplementary materials

Table S1. The ECG-based individual cardiac function biomarkers in resting and cold pressor conditions

		**Resting**							**Cold pressor**							
	**ID number**	**RR-interval (ms)**	**HR (bpm)**	**LF (ms^2)**	**HF (ms^2)**	**LF/HF**	**DC (ms)**	**PRD (degree)**	**RR-interval (ms)**	**HR (bpm)**	**LF (ms^2)**	**HF (ms^2)**	**LF/HF**	**DC (ms)**	**PRD (degree)**	**Endurance time (s)**
RR-interval in rest state > RR-interval in cold pressor state	ID1009	746.09	80.62	619.82	334.25	1.85	10.07	0.44	696.17	86.52	40.61	392.22	0.10	9.51	0.39	27
	ID1010	915.68	65.68	301.36	1353.77	0.22	18.81	0.36	823.80	73.10	364.79	852.90	0.43	16.13	0.45	122
	ID1002	787.99	76.25	169.77	101.30	1.68	6.26	0.35	783.71	76.88	401.34	99.11	4.05	7.30	0.44	119
	ID1004	796.25	75.65	1200.43	356.03	3.37	13.93	0.40	738.08	81.64	8.87	382.75	0.02	11.46	0.50	43
	ID1005	812.55	74.05	540.39	879.08	0.61	18.01	0.39	741.97	81.34	1127.28	422.30	2.67	16.16	0.52	78
	ID1007	780.43	76.99	241.20	221.42	1.09	7.90	0.36	757.20	79.42	471.11	332.51	1.42	9.71	0.29	96
	ID1008	670.35	89.90	272.14	677.09	0.40	8.66	0.44								
	ID1006	826.01	73.11	1482.27	1539.94	0.96	22.08	0.44	764.71	78.96	820.36	746.12	1.10	15.67	0.42	121
	ID2003	744.04	80.82	578.80	270.01	2.14	10.85	0.46	697.70	86.31	3.77	45.94	0.08	11.08	0.63	20
	ID2007	864.04	69.88	1183.15	1324.69	0.89	23.47	0.35	831.08	72.91	1662.22	1991.42	0.83	28.12	0.41	100
	**Mean**	**794.34**	**76.30**	**658.93**	**705.76**	**1.32**	**14.00**	**0.40**	**759.38**	**79.68**	**544.48**	**585.03**	**1.19**	**13.90**	**0.45**	**80.3**
	**S.E.**	**20.34**	**1.99**	**139.97**	**160.62**	**0.29**	**1.86**	**0.01**	**14.33**	**1.47**	**168.54**	**175.46**	**0.41**	**1.86**	**0.03**	**11.6**
	**p-value**	**0.001**	**0.000**	**0.462**	**0.410**	**0.717**	**0.529**	**0.081**								
RR-interval in rest state< RR-interval in cold pressor state	ID2004	856.18	70.29	536.58	113.09	4.74	7.47	0.40	879.89	68.91	251.76	747.42	0.34	14.48	0.40	44
	ID2006	788.17	76.48	544.09	1065.96	0.51	17.55	0.27	880.90	68.56	1745.91	2077.74	0.84	26.16	0.43	122
	ID2005	776.39	77.60	793.05	638.74	1.24	15.08	0.31	802.69	75.23	1823.42	967.23	1.89	20.72	0.30	130
	ID2008	784.62	76.90	488.48	474.19	1.03	13.31	0.31	824.54	73.05	853.88	644.44	1.32	15.84	0.37	132
	ID2009	827.67	72.84	764.89	1140.31	0.67	19.58	0.38	857.01	70.44	830.66	2182.89	0.38	24.79	0.35	113
	ID1003	822.89	73.08	341.59	284.42	1.20	8.86	0.42	857.27	70.08	12.13	134.61	0.09	5.88	0.45	32
	ID1011	801.02	75.68	2282.65	1336.41	1.71	20.73	0.30	848.86	71.27	2638.33	2283.76	1.16	25.80	0.39	131
	**Mean**	**808.13**	**74.70**	**821.62**	**721.87**	**1.59**	**14.65**	**0.34**	**850.17**	**71.08**	**1165.16**	**1291.16**	**0.86**	**19.10**	**0.38**	**100.5**
	**S.E.**	**10.02**	**0.94**	**232.04**	**162.97**	**0.51**	**1.79**	**0.02**	**9.94**	**0.83**	**329.30**	**304.91**	**0.22**	**2.62**	**0.02**	**12.7**
	**p-value**	**0.003**	**0.004**	**0.178**	**0.018**	**0.309**	**0.021**	**0.138**								

Notes: S.E., Standard error, p-value, results of the paired student t-test between resting and cold pressor conditions.
HR: Heartbeat rate; LF: Low-frequency power (0.04-0.15 Hz); HF: high-frequency power (0.15-0.4 Hz); DC: Deceleration capacity of heart rate; PRD: Periodic repolarization dynamics. ID1XXX: ID number of the participants from the Asian Institute of Gastroenterology, Hyderabad, and ID2XXX: ID number of the participants from the All India Institute of Medical Sciences, New Delhi.
